# Experiments on Animals Relative to the Question of Abdominal Supporters after Laparotomy1Read at the twenty-first annual meeting of the American Association of Obsteticians and Gynecologists at Baltimore, pbe 1909.

**Published:** 1909-11

**Authors:** Robert T. Morris

**Affiliations:** New York


### Experiments on Animals Relative to the Question of Abdominal Supporters after Laparotomy.[1]

By ROBERT T. MORRIS, M. D., New York.

(*From American Journal of Obstetrics.*)

FOR a good many years I have made it a rule not to apply an abdominal supporter after abdominal operations, relying mainly on methods of suturing. Now and then a patient will come into my office wearing an abdominal supporter asking me if she cannot take it off. In reply I ask the patient who put it on, and generally the answer is that her doctor or nurse, or some friend recommended it. Friends are apt to interview patients after operations and induce them to invest money in abdominal

1. Read at the twenty-first annual meeting of the American Association of Obstetcians and Gynecologists at Baltimore, pbe 1909.

supporters. These supporters are, for the most part, abominable, being of no earthly use. Abdominal supporters, as I see them, are not doing any good whatsoever. The *vis a tergo* of a progressive hernia is not restrained or relieved by an abdominal supporter, and it has been my rule not to apply them at all.

In repairing the abdominal wall, I have made it a rule not to employ any fanciful methods of closure, but simply try to leave things as I found them. If the different layers of tissue are brought together properly, healing will take place. It is very essential to get the muscle margins and the skin margins together; in other words, to leave things as they were found.

In making a number of experimental tests upon animals to determine the length of time required for repair, I was interested in the results. For example, I experimented with a series of rabbits, the rabbits being put in the horizontal position. Eight or ten rabbits were operated on. At the end of three days we, I and my assistant, cut out a strip of tissue three inches long and half an inch wide, one including the line of incision through the abdominal wall. Another similar strip was cut out, but we had to leave part of the abdominal wall undisturbed, fastening one end of the strip with a pair of clamps, and the other being pulled on with forceps. The first rabbit was killed within three days. The strip of tissue that was normal, not including the incision, gave this result: the skin tore apart at eighteen pounds pull; the muscle and fascia pulled apart at sixteen pounds pull; the peritoneum gave away at seven pounds pull. The sutured area in this rabbit, killed three days after operation, did not give resistance sufficient to stand one pound pull on any structure. Another rabbit, killed seven days after operation, gave this result: normal skin pulled apart at seventeen pounds pull; skin pulled apart at two pounds; normal muscle and fascia pulled apart at fourteen pounds; sutured muscle and fascia pulled apart at five pounds; normal peritoneum pulled apart at eight pounds; sutured peritoneum pulled apart at eight pounds.

This is interesting as showing the relative degree of repair observed in this series as well as in a number of other animals. The peritoneum was practically entirely repaired at the end of seven days, while the other structures underwent repair less slowly, and the skin last of all as shown in my experiments.

Another rabbit, killed ten days after operation, showed that repair was perfect practically in all structures, but the muscle tear extended into the stitch holes, or the tear extended into normal tissue alongside. The tissue gave way at the skin wound line only at the end of ten days. In a rabbit, killed fourteen days after operation, as I pulled one could see the fibers slit, and not one of the structures gave way at the wound line at the end of fourteen days, excepting as it took a part of the normal structures. In the skin one could see the different fibers stretching during the pull. One could observe the fibers of the muscle and fascia slitting as in a textile fabric. The peritoneum of the rabbit is adherent, but you can pinch it up with your fingers. At the end of fourteen days all structures were repaired in the rabbits. Rabbits killed eighteen, ten, twenty-one and thirteen days after operation, all gave similar results.

The important point I wish to make is this: most of the abdominal supporters applied after operation are applied unnecessarily, and practitioners do not have in mind the proper idea of the mechanics involved, even though patients are in a perpendicular position most of the day instead of the horizontal posture.

There is one special point I wish to make and that is this: in suturing the layer of skin or fascia, I am very careful not to allow any suture to get into the layer of fat, because it will allow some particles of fat to go through, and if any should go through it is bad for us. The suture goes through skin only. If the patient has a layer of fat four inches thick, not a particle of that suture is allowed to go into any part of that fat, and I am very, very careful not to allow the needle-point to go into that fat. No fat is lost. By suturing skin only and muscle layer beneath, leaving the fat entirely without suture, we get the effect of atmospheric pressure. This atmospheric pressure, from a mechanical standpoint, is of vast importance, and, it seems to me, we should strive to restore the abdominal incision in such a manner as to do away with these pink ornaments, these elastic abdominal supporters, which are odoriferous, as well as useless.

616 Madison Avenue.

